# Evidence of collective influence in innate sensing using fluidic force microscopy

**DOI:** 10.3389/fimmu.2024.1340384

**Published:** 2024-01-23

**Authors:** Elizabeth J. Mulder, Brittany Moser, Jennifer Delgado, Rachel C. Steinhardt, Aaron P. Esser-Kahn

**Affiliations:** Esser-Kahn Lab, Pritzker School of Molecular Engineering, University of Chicago, Chicago, IL, United States

**Keywords:** NF-κB, macrophage, RAW 264.7, fluidic force microscopy, FluidFM, quorum licensing, resiquimod, innate immunity

## Abstract

The innate immune system initiates early response to infection by sensing molecular patterns of infection through pattern-recognition receptors (PRRs). Previous work on PRR stimulation of macrophages revealed significant heterogeneity in single cell responses, suggesting the importance of individual macrophage stimulation. Current methods either isolate individual macrophages or stimulate a whole culture and measure individual readouts. We probed single cell NF-κB responses to localized stimuli within a naïve culture with Fluidic Force Microscopy (FluidFM). Individual cells stimulated in naïve culture were more sensitive compared to individual cells in uniformly stimulated cultures. In cluster stimulation, NF-κB activation decreased with increased cell density or decreased stimulation time. Our results support the growing body of evidence for cell-to-cell communication in macrophage activation, and limit potential mechanisms. Such a mechanism might be manipulated to tune macrophage sensitivity, and the density-dependent modulation of sensitivity to PRR signals could have relevance to biological situations where macrophage density increases.

## 1 Introduction

The innate immune system is responsible for early response to infection or vaccination by detecting molecular patterns from pathogens and then activating surrounding and downstream immune cells. In the earliest stages of the response, individual immune cells play a role in detecting pathogen-associated molecular patterns (PAMPs), most notably molecules that activate Toll-Like Receptor (TLR) pathways (1).

Many cells signal and sense via complex dynamics depending on the temporal behavior of the stimulus and factors in the cellular environment (2–9). Methods that stimulate cells uniformly and measure individual responses have found heterogeneity in single immune cell responses to TLR stimulation (10). However, few experiments have probed immune cell responses with localized PRR stimulation instead of constant, uniform stimulation. In this paper we use Fluidic Force Microscopy (FluidFM) to localize the stimulus down to the single-cell level, allowing for exquisite spatiotemporal control of single cell stimulation, as well as providing a platform for monitoring the downstream effects of stimulation. By moving to single cell stimulation and readout we can further investigate these heterogeneities (11, 12).

Current methods of studying single immune cells involve either isolation or stimulation of a whole culture and the subsequent measurement of individual cell readouts. We turned to techniques employed in neurobiology (such as the stimulation of single neurons in a network) for options. Technologies including optogenetics, photocaged molecules, and specialized micropipettes have been used to briefly stimulate individual cells (13–18). However, immune cell PRR activation requires stimulation from minutes to hours, so single immune cell stimulation requires a method that can provide sustained stimulation at one precise location (19). Fluidic Force Microscopy (FluidFM) builds upon micropipette techniques with the addition of an AFM-based controller, that holds and positions the micropipette probe with micrometer precision to ensure accurate levels of agonist are delivered (20). FluidFM technology has been used to measure individual cell responses to local chemical stimulation (20, 21). It allows for sub-millimeter scale perturbations in a cell’s environment while preserving the rest of its environmental context, allows for monitoring over the length and timescales relevant to macrophage activation, and gives the user precise control over the location, concentration, and duration of stimulation.

In applying this FluidFM technology to study single immune cell responses, we chose TLR-induced NF-κB activation of RAW 264.7 monocyte/macrophage-like cells as a model system. TLR-induced NF-κB translocation is commonly used as a readout of innate immune activation via TLR pathways and RAW 264.7 cells are commonly used as an *in vitro* model of macrophage responses (9, 22–25). Macrophages play a key role in the early immune response to infection or vaccination by releasing chemical signals to initiate an inflammatory immune response in response to TLR stimulation. Uniform TLR stimulation of macrophages has revealed significant heterogeneity in single immune cell responses (10, 11, 26, 27), suggesting the importance of studying individual macrophage stimulation. Macrophages are well suited for use with FluidFM technology: they are adherent, they respond to a soluble ligand dispensed by the FluidFM, and the timescales of their NF-κB activation and response readily provide clear measurements via fluorescent microscopy of multiple cells allowing for sufficient datasets for statistical analysis. While NF-κB activation is only one early indicator of macrophage activation, it provided a fast, live, single-cell readout for these experiments. Using this unique tool and set of experimental conditions, we sought to understand how individual macrophages respond to a local stimulus while surrounded by unstimulated cells. By comparing these values, we report on a role for cell density in determining how individual macrophages mediate a collective response.

## 2 Materials and methods

### 2.1 RAW 264.7 NF-κB reporter monocyte/macrophage-like cell line

RAW 264.7 monocyte/macrophage-like cells (RRID: CVCL_0493, ATCC TIB71) derived from BALB/c mice, mouse leukemia, immortalized cell line, sex of cell male. Reporter cell line constructed by Sung et al. (28) and obtained from the Fraser lab. Growth conditions: cultured in DMEM (sterile filtered, Thermo Fisher 11995073) with 10% FBS (sterile, Thermo Fisher 26140079) in a biological safety cabinet, grown at 37°C and 5% CO_2_ in a sterile incubator and used prior to day 30.

### 2.2 Cell culture preparation

Reporter RAW 264.7 cells were plated in 2 mL DMEM with 10% FBS in a 50 mm microscope dish (TedPella 14027-200) 1-2 days before the experiment (200,000 - 500,000 cells depending on desired final density) or cultured in 8-well chamber slides (Ibidi 80826 or Thermo Fisher 155411) at 100,000 cells per well. One hour before the experiment, media was changed to 10% HIFBS (Thermo Fisher 16140071) in CO2-Independent media (Gibco, Fisher Scientific 18045088) with 2% L-glutamine (for live cell microscopy) or 10% HIFBS in DMEM (for incubator experiments). The cells were then incubated 30-60 min at 37°C and atmospheric CO_2_ (on the microscope) or 5% CO_2_ (in the incubator), stained with 100 ng/mL hoechst 33342 nuclear stain (Fisher Scientific H3570), and incubated a further 15-30 min. Cells were kept in the incubator or heated microscope box (37°C) for the duration of the experiment. Cells were stimulated with Resiquimod (R848, Invivogen tlrl-r848). R848 was selected based on the requirement for a small molecule to provide even flow through the small opening in the FluidFM probe.

### 2.3 Microscopy

Imaging was performed using a Zeiss Axio Observer 7 inverted optical microscope, Hamamatsu ORCA-Flash4.0 V3 sCMOS camera (Hamamatsu C13440-20CU-KIT), Spectra-X Light Engine, and Zeiss Zen Pro software. Microscope equipped with Pecon live cell incubation box, Zeiss Heating Unit XL S, and Zeiss TempModule S to maintain temperature at 37°C when imaging live cells. Reporter RAW 264.7 cells in chambered slides were imaged using a 10x air objective (Zeiss 420640-9900-000). Reporter RAW 264.7 cells stimulated with the FluidFM were imaged at the same location before and after treatment using a 10x or 100x (Zeiss 421090-9800-000) air objective. Channels: hoechst33342 nuclear stain (Ex/Em 350/461) and GFP reporter (Ex/Em 488/507).

### 2.4 Whole culture stimulation: method development

Development of protocol for whole culture stimulation with stimulation time less than readout time. Both the CO_2_ independent medium and the DMEM media in the incubator yielded similar NF-κB activation (**Supplementary Figure 1A**). Next, three methods of agonist addition were tested: dilution of agonist into the cell supernatant, replacing cell supernatant with agonist in conditioned media, and replacing cell supernatant with agonist in fresh media. The dilution method yielded higher activation than either replacement method (**Supplementary Figure 1B**). Lastly, two agonist removal methods were tested: PBS wash (removing well contents, washing twice with warm PBS, then replacing with warm, untreated media) and dilution wash (removing 250 μL the 300 μL well contents, leaving 50 μL to cover the cells and prevent them drying, adding 250 μL fresh, warm media to dilute the agonist, and repeating 4 times to ensure agonist is diluted below activating levels; total dilution approx. 1:1000). Removing the agonist by dilution yielded higher activation than removing the agonist by PBS wash, and for 15-minute stimulation we compared the dilution removal method with no wash (readout immediately at 15 minutes), and the activity was preserved (**Supplementary Figure 1C**). We attribute the lower activation with PBS wash to the disturbance caused by removal of complete cell contents, compared to the gentler dilution wash, so this method is preferred for short stimulation times where the agonist needs to be removed before NF-κB activation can be read out.

### 2.5 Whole culture stimulation in incubator

Whole culture experiments were conducted using media, PBS, and agonist dilutions in media prewarmed to 37°C in the incubator. Untreated cell supernatant was taken from a culture flask plated and prepared alongside the chambered slides (Ibidi 80826). One hour before the experiment, the media in the wells was changed to DMEM with 10% HIFBS and hoechst stain. At the start of the experiment, cells were treated by diluting the agonist 1:10 in the wells and mixing by pipette and rocking, then incubating at 37°C and 5% CO2 for the indicated stimulation time, diluting the well contents 4 times at 1:6 ratio with warm, untreated media, then incubating further for a total of 30 minutes incubation time. Cells were washed with warm PBS and fixed with Cytofix (BD Biosciences 554655) at 4°C. Fixed cells were washed once more and imaged in PBS. For live-cell imaging, instead of fixing the cells were imaged immediately at 15 minutes.

### 2.6 Whole culture stimulation for density comparison on microscope

Whole culture stimulation and density comparison experiments were conducted using reporter RAW 264.7 cells plated at 375 and 3,750 cells/mm^2^ in 8-well chamber slides (Thermo Fisher 155411) and grown overnight at 37°C and 5% CO_2_ in the incubator. One hour before treatment, the media was changed to 300 μL/well CO2 Independent Medium with 2% L-glutamine, 10% HIFBS, and 100 ng/mL hoechst stain, and the cells incubated at 37°C. Cells were stimulated with R848 (Invivogen tlrl-r848), Lipopolysaccharide (LPS-EB Ultrapure, Invivogen tlrl-3pelps), or Lipoteichoic Acid (LTA) (Invivogen tlrl-lta) for 5 minutes, washed by serial dilution as described above, then incubated for 10 minutes and imaged (hoechst and GFP as described above).

### 2.7 FluidFM dispensing

In the FluidFM experiments, cells were treated by dispensing agonist out of a micropipette probe (2 μm aperture, 2 N/m stiffness, FlexAFM compatible, Cytosurge), positioned by the Flex AFM FluidFM controller (Nanosurf) using the Cytosurge software interface (Cytosurge). The target cell or spot was positioned in the center of the field of view using the motorized stage control. Then, the probe was approached to the surface of the dish (in a bare spot), with contact detected by the optical feedback system of the FluidFM, and then retracted by 4 or 20 μm (for single cell or cluster stimulation, respectively) using the software controls. The probe opening was positioned over the target cell (single-cell stimulation) or the center of the frame (cluster stimulation) using the manual control which moves the controller (and thus the probe attached to it) in the horizontal plane, separate from the culture dish. The probe was cleaned between each experiment with NovoRinse (Agilent 872B603) and NovoClean (Agilent 872B602) followed by sterile cell culture water. More details of the FluidFM Protocol can be found in (Mulder et. al, STAR Protocols, in press).

### 2.8 Flow cytometry

RAW264.7 cells were cultured at high and low density (2500 and 400 cells/mm^2^ respectively) overnight in a 6-well plate (CellTreat 229106) in DMEM media supplemented with 10% FBS. Cells at each density were divided into five aliquots of equal cell count (approximately 0.5 x 106) and washed with PBS, stained with a live dead reactive dye (BioLegend L34975), fixed and permeabilized with Cyto-Fast Fix/Perm Kit (BioLegend 426803) following kit instructions, and then blocked with anti-mouse CD16/32 antibody (BioLegend 101319) at 10 ug/mL. For each density, three samples were stained with PE anti-mouse CD287 (TLR7) Antibody (BioLegend 160003) at 5 ug/mL, one with PE Mouse IgG1, κ Isotype Ctrl (FC) Antibody (BioLegend 400113) at 5 ug/mL, and one left unstained, at the steps indicated in the Fix/Perm protocol. The cells were then washed and resuspended in Cell Staining Buffer (BioLegend 420201) and run on an ACEA NovoCyte Flow Cytometer.

### 2.9 Calibration data for quantification

Quantified calibration data of resting and activated RAW 264.7 cells was obtained following the method described by us previously (Mulder et. al, STAR Protocols, in press), and cutoff values for activation determined for each experimental setup (**Supplementary Figures 1D-H**). Briefly, images were taken of resting and activated RAW 264.7 cells under the same conditions as for each experiment (**Supplementary Figure 1D**), quantified to determine the nuclear/cytoplasmic NF-κB ratio of each cell (**Supplementary Figure 1E**), and cutoffs set at the top 5% of the resting population (**Supplementary Figures 1F–H**). These cutoffs were used to determine the fraction of cells activated above background levels for each experiment. NF-κB activation was imaged at 15 minutes post-stimulation, to capture the initial peak of activation (**Supplementary Figure 1I**). The distribution of nuclear/cytoplasmic NF-κB ratio values for resting cells remained stable over time (**Supplementary Figures 1J, K**).

### 2.10 Quantification and statistical analysis

Cell images were quantified with CellProfiler (RRID : SCR_007358) (29) for single-cell intensity measurements. Cell tracking and segmentation errors were corrected manually for the time-series data. Statistical analysis, curve fits, and plots were produced using GraphPad Prism (RRID : SCR_002798), Mathematica (30), and RStudio (RRID : SCR_000432) (31). Statistical significance and effect size determined as described in figure captions, by Wilcoxon test in R or bootstrap statistical methods. Number of neighbors calculated as the number of other cell nuclei within 18 μm of each cell nucleus (determined empirically) using custom code in R. Flow cytometry data were analyzed and plotted in R using the flowCore (RRID : SCR_002205) and flowViz packages (32, 33). All custom code is available on Mendeley Data, DOI: 10.17632/mfyddz6n8k.3.

## 3 Results

### 3.1 Spatial and temporal control of TLR agonist to target single, clusters, and whole cultures of RAW 264.7 cells

**Figure 1:**
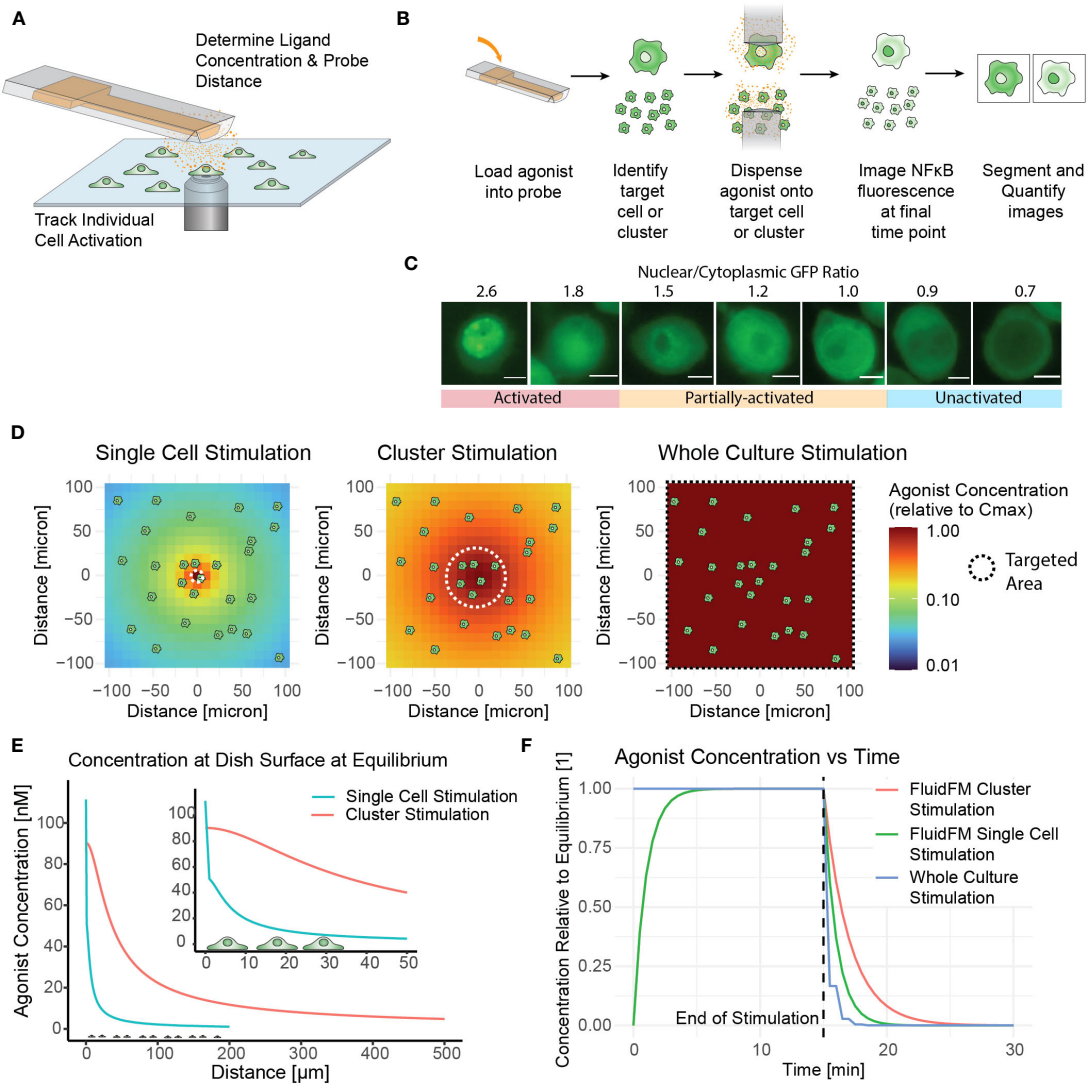
Fluidic Force Microscopy allows for stimulation and monitoring of single cells and clusters in culture. **(A)** Schematic of FluidFM experiment showing probe, cells, diffusing agonist, and microscope objective. **(B)** FluidFM experimental workflow for single cell and cluster targeting with live-cell NF-κB response readout. **(C)** Quantified images of NF-κB readout, scale bar 5 μm, activation numerical cutoff from calibration data (**Supplementary Figure 1G**). **(D-F)** Spatiotemporal parameters of agonist concentration based on model of agonist diffusion (Mulder et. al, STAR Protocols, in press). Cartoon cells shown for scale. **(D)** Schematic of concentration vs distance for FluidFM single cell and cluster stimulation and whole culture stimulation, shown from an overhead view of the cell culture. Color indicates agonist concentration relative to the maximum in the center (C_max_). **(E)** Side view for the condition where C_max_ is closest to 100 nM for each mode (C_0_ = 5.5 μM for single cell stimulation and 0.318 μM for cluster stimulation). **(F)** Time-course of agonist concentration in FluidFM dispensing and whole culture stimulation experiments; 15-minute stimulation time shown as an example.

Although the NF-κB activation of RAW 264.7 cells in culture and in isolation has been studied, the situation of targeted activation of one or a few cells in culture remained elusive. Our initial goal was to determine the concentration of ligand needed to activate a single macrophage in culture using TLR7/8 as a model agonist – a threshold of activation. To accomplish this, we delivered agonist (Resiquimod, or R848) directly onto the target cell(s) in pico-liter volumes using the FluidFM (**Figures 1A, B**). This technology allowed us to stimulate a single cell within a larger microenvironment composed of unstimulated cells. We monitored the cells throughout with live cell fluorescence microscopy (**Figures 1A-C**). Subsequently we developed methods for cluster stimulation in culture, and whole culture stimulation, of the same stimulation times to compare with the single cell stimulation results. These three methods allowed us to target the RAW 264.7 cells on three different scales and compare the response (**Figure 1D**, **Table 1**).

**Table 1:** Methods comparison: stimulation area and number of cells.

	Single Cell	Cluster	Whole Culture
*Width of Area Stimulated*	10^1^ μm	10^1^ – 10^2^ μm	1 cm
*Number of Cells Stimulated*	10^0^ cells	10^1^ – 10^2^ cells	10^4^ – 10^5^ cells

Order of magnitude estimates for width of area and number of cells stimulated with each of the three methods: single cell stimulation, cluster stimulation, and whole culture stimulation.

To assess the macrophage immune response, we employed a commonly used measure of macrophage activation – nuclear translocation of transcription factor NF-κB. In the resting state, NF-κB resides in the cytoplasm, and translocates to the nucleus after TLR activation (1). Using the FluidFM (20, 21, 34, 35), we treated individual RAW 264.7 NF-κB reporter cells (28) in culture with a localized stimulus and monitored the NF-κB response by tracking the translocation of the GFP reporter (**Figures 1A–C**, **Supplementary Figures S1D, E**) (19). We then categorized the single cell responses with calibration data to establish a quantitative activation cutoff and determine the fraction activated for each set of experimental parameters (**Figure 1C**, **Supplementary Figures S1F–H**).

Having established methods for treating and monitoring single cells in culture, we then determined stimulation conditions. Several variables were considered in the design of this study, including cell density, agonist type, agonist concentration, stimulation time, and time at image collection. To simulate physiological macrophage densities, we plated RAW 264.7 cells within the range estimated for tissue-resident macrophages: <1000 cells/mm^2^ (**Supplementary Figure 1L**) (36, 37). We also plated RAW 264.7 cells at more confluent densities for comparison. For the agonist we selected the TLR7/8 agonist R848 for three reasons. First, R848 dispenses well out of the FluidFM probe without aggregating or clogging. Second, as a small molecule its diffusion could be mathematically modeled for an accurate concentration gradient. Finally, it consistently elicited measurable NF-κB activation in 15 minutes (**Supplementary Figure 1I**), allowing for rapid collection of single-cell datasets (19).

In addition to the minimum response time (15 min), we tested shorter stimulation times until we established a minimum stimulation time (5 min) that elicited a response. For single cell stimulation without making physical contact, we dispensed ligand from the 2-micron diameter probe opening at a minimal flow rate a few microns above the targe cell surface, and for cluster stimulation we dispensed at a greater height and flow rate to cover a wider area with a more uniform concentration (**Figures 1D, E**). The ligand then diffused rapidly in the area surrounding the target cell until establishing a stable concentration gradient where agonist diffusing away from the area was replaced by fresh agonist dispensed from the probe (**Figures 1D-F**). After removal of the FluidFM probe, the agonist would diffuse away from the stimulated area within minutes (**Figure 1F**). The concentration of R848 loaded into the probe (C_0_) and the position and dispensing rate of the probe together determine the concentration gradient of agonist at the dish surface (**Figures 1C, D**). C_max_ denotes the concentration at the center of the gradient, right below the dispensing point. The concentration of agonist at the position of each cell is a function of C_0_, the dispensing conditions, and the diffusion of the agonist into the surrounding media over time. The spatiotemporal dynamics of the concentration gradient under FluidFM dispensing were characterized as described by us previously (Mulder et. al, STAR Protocols, in press).

To compare with single cell and cluster stimulation, we designed a short-timescale-stimulation version of a conventional dose curve to measure NF-κB activity. The most challenging part of this was that the shortest stimulation time (5 minutes) was less than the minimum response time for the reporter cells (15 minutes) (19) (**Supplementary Figure 1I**), meaning we could not simply stimulate the cells for 5 minutes and image right away – we needed a minimally-disruptive method to remove the agonist and continue incubating. We tested several methods of adding and removing the agonist from the culture media (**Supplementary Figures 1A-C**). We selected the method that had the highest NF-κB response at the shortest stimulation times: serial dilution with warm media (**Supplementary Figure 1C**).

The combination of single cell targeting, cluster targeting, and whole culture stimulation methods allowed us to target areas and numbers of cells across five orders of magnitude (**Table 1**) and determine the minimum concentration and stimulation times that yielded NF-κB activation in single cells, small clusters, and whole cultures. By comparing single and collective responses, we could investigate the influence of neighboring cells on an individual cell or cluster response.

### 3.2 Single cells activate with a lower concentration stimulus than a culture of the same cells

**Figure 2:**
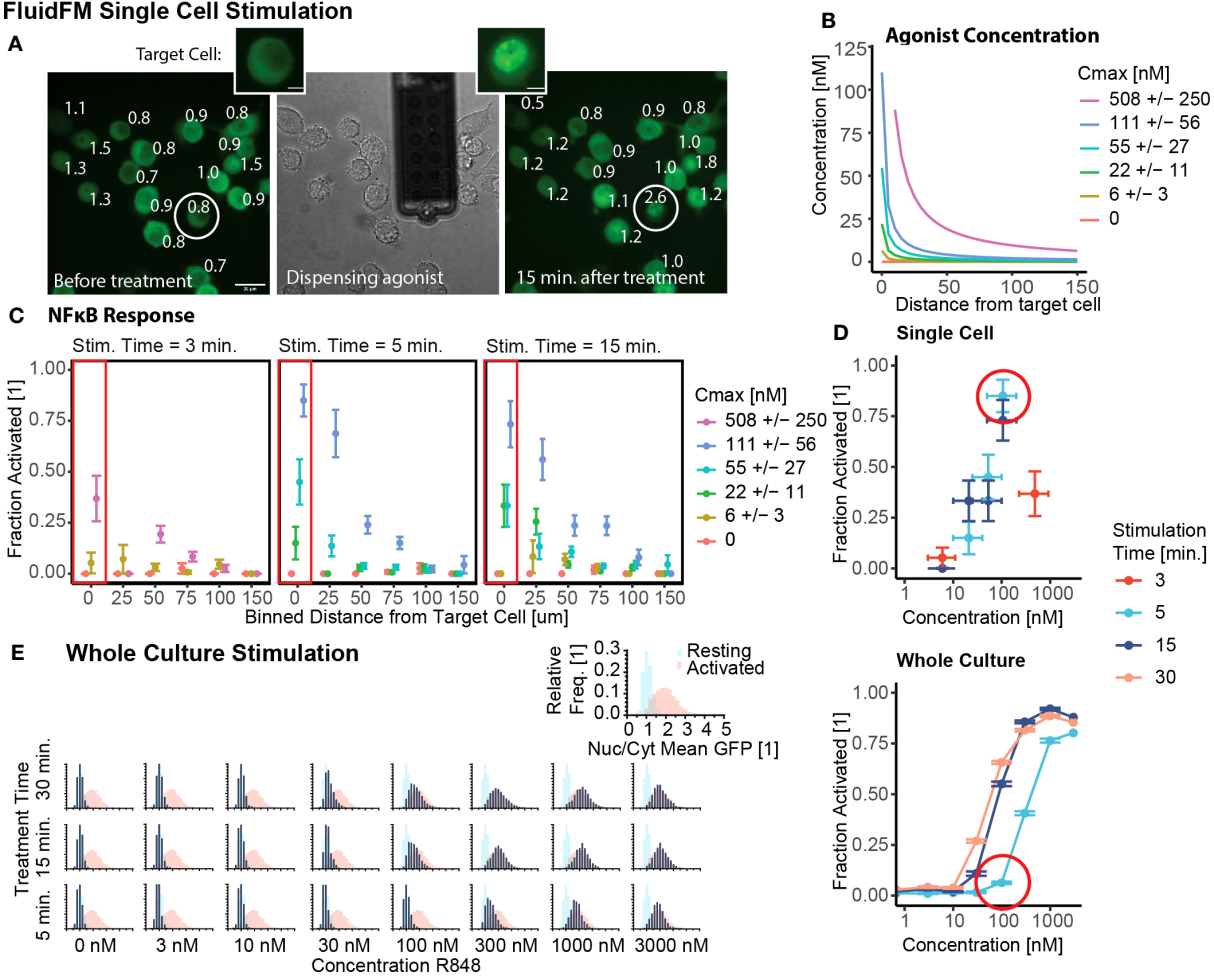
Single cells activate with lower concentration stimulus than a culture of the same cells. **(A)** Example of FluidFM experiment targeting a single cell. Left to right: GFP fluorescence before dispensing, brightfield image during dispensing, GFP fluorescence 15 min. after dispensing. Target cell circled, numbers are Nuclear/Cytoplasmic GFP ratio. Scale bar 20 μm. Inset: target cell before, after stimulation, scale bar 5 μm. **(B)** Agonist concentration vs distance for each single cell stimulation condition, colors are concentration at target cell position (C_max_). **(C)** Fraction activated (measured at 15 minutes for 3 and 5 minute stimulation, measured at 30 minutes for 15 minute stimulation) vs distance from dispensing point for each stimulation condition shown in **(B)**, stimulation times (left to right) are 3, 5, and 15 minutes. Each group has 15-21 independent replicates (**Supplementary Table 1**). **(D)** Fraction activated vs concentration, stimulation time from **(C, E)**. Top: target cells in single cell stimulation [boxed in **(C)**]. Horizontal error bar uncertainty in concentration at target cell position (Mulder et. al, STAR Protocols, in press). Bottom: whole culture stimulation. Vertical error bars are uncertainty in fraction activated, calculated by the bootstrap method (Mulder et. al, STAR Protocols, in press). **(E)** Group stimulation in chambered slides. Calibration data (**Supplementary Figure 1F**) of resting (blue) and activated (red) populations in each panel. Data in navy, all scales same as inset. Histograms arranged by R848 concentration and treatment time.

We started by investigating the conditions with the greatest contrast: single cell stimulation and whole culture stimulation. We tested both the concentration and temporal limits of single cell sensitivity with the FluidFM. First, we confirmed that all cells were in resting state before the start of each experiment (**Supplementary Figures 1K**, **2A**), and that single cell stimulation with FluidFM reliably activated single cells in culture (**Figure 2A**). Next, using the concentration calculations for the FluidFM (**Figures 1D, E** and **2B**) (Mulder et. al, STAR Protocols, in press), we stimulated single RAW 264.7 cells with R848 concentrations from 6 to 500 nM for 3, 5, and 15 min. and analyzed the response of the target and surrounding cells. The response is highest for the target cell and decreases with increasing distance from the target (**Figures 2A, C**, **Supplementary Figure 2B**). We compared concentration at the target cell position (**Figure 2B**) with activation (**Figure 2C**) resulting in a target cell dose curve (**Figure 2D**, **Supplementary Table 1**). The results demonstrated that stimulation with R848 at a concentration of ~100 nM for 5 min was sufficient to activate the majority of RAW 264.7 cells individually. (There is about 50% uncertainty in C_max_ for the single cell stimulation experiments, depending on the distance between the cell surface and the probe; as cell height is variable, this concentration cannot be more precisely known) (Mulder et. al, STAR Protocols, in press). An activating concentration of 100 nM is consistent with previous reports of NF-κB activation by R848 stimulation, however the 5-minute timing is considerably shorter than most stimulation times for TLR agonists.

We next used our short-timescale whole culture activation protocol to investigate the whole culture response at that same concentrations and stimulation times as the single cell experiments. (**Figure 2E**, **Supplementary Figure 2C**). The NF-κB response of the population increases from a resting state to an activated state with increasing concentration and/or stimulation time. Stimulation at or above 100 nM for 15+ minutes is sufficient to cause activation, but for 5-minute stimulation, the concentration threshold is tenfold higher (**Figures 2C, D**).

These results provide context for the single cell stimulation FluidFM results. The minimum concentration for activation with any stimulation time (100 nM) is nearly the same in both experiments. However, in the case of short stimulation time (5 minutes), the whole culture activation threshold is higher than the single cell threshold. Using a permutation analysis (Mulder et. al, STAR Protocols, in press), random samples were drawn with replacement from the combined data and the fraction activated calculated. When compared with the experimental result for the single cell activation, none of the 5000 sampled datasets had as high of a fraction activated (**Supplementary Figure 2D**). Whole culture stimulation compared to single cell stimulation decreased the percent activated by 79% +/- 8% (85% to 6%). From these results we concluded that the activation threshold for R848 is lower for single cells than a culture of cells.

### 3.3 Increase in local cell density leads to decrease in activation, suggesting a role for cell-to-cell communication

**Figure 3:**
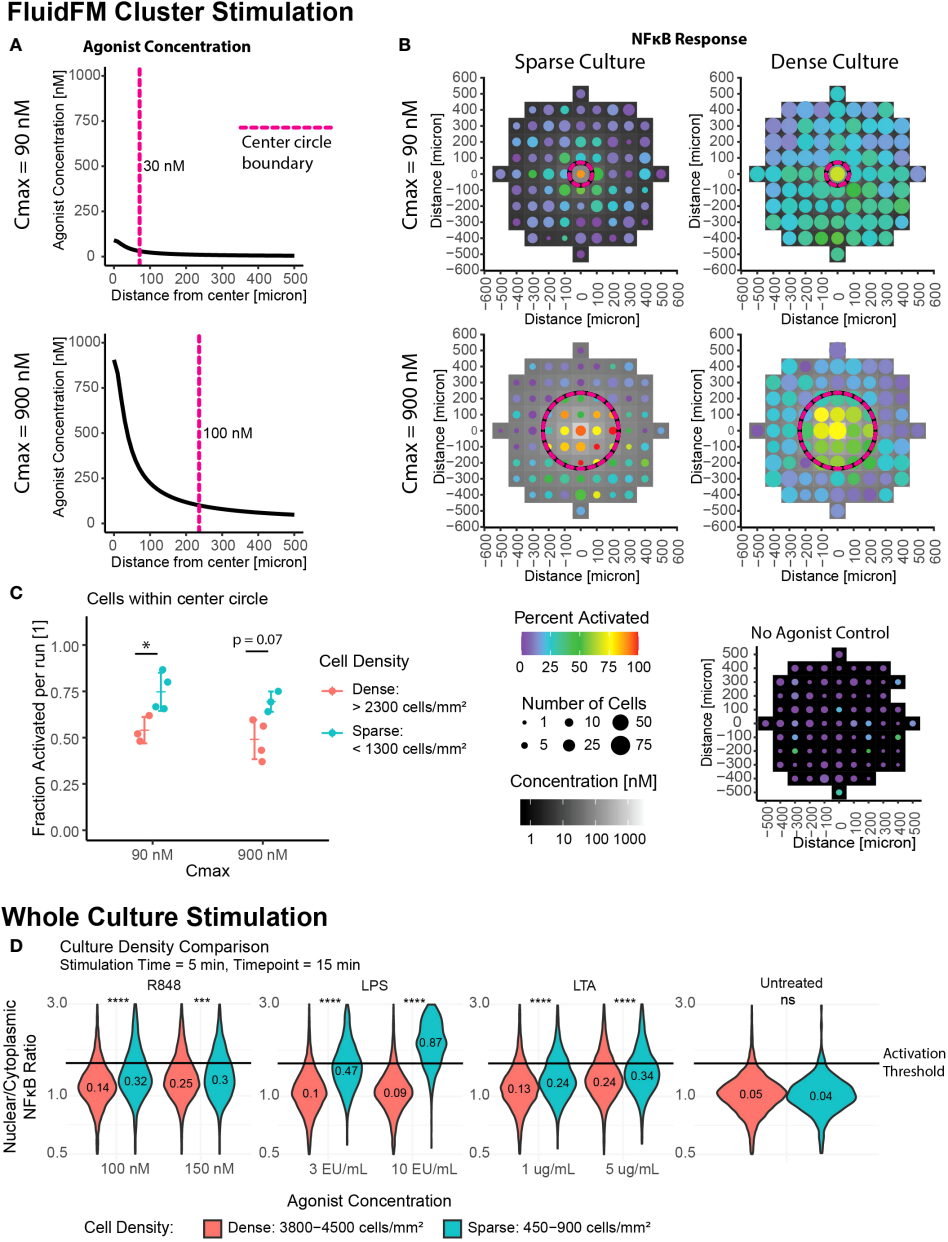
Increase in local cell density leads to decrease in activation, suggesting role for cell-to-cell communication. Cluster stimulation with FluidFM at different cell densities. **(A)** Concentration gradient during cluster stimulation for C_max_ = 90 nM (top) and 900 nM (bottom). Dashed line marks where concentration drops below 30 nM (top) or 100 nM (bottom), cutoffs chosen based where activation drops below 50% for that stimulation condition (**Supplementary Figure 3A**). **(B)** Cluster stimulation with FluidFM, each plot one representative experiment (**Supplementary Figure 4A**). Top row C_max_ = 90 nM, bottom C_max_ = 900 nM, left column sparse plating, right dense plating, all 5 min. stimulation time. Data binned in 100 μm x 100 μm squares, tile background is agonist concentration, point color is percent activated and point size is the number of cells per bin. Circular dashed line corresponds to dashed line in **(A)**. Negative control (bottom right). **(C)** Fraction activated in center per independent experiment, cutoff from calibration data (**Supplementary Figure 1H**). Grouped by stimulation condition (Cmax) and cell density. Significance from permutation ANOVA in R (Mulder et. al, STAR Protocols, in press). **(D)** Whole culture activation: culture density comparison. Reporter RAW 264.7 cells treated with LPS, LTA, or R848 at the indicated concentrations for 5 minutes, agonist removed by dilution method, incubated for a further 10 minutes, then imaged. NF-κB translocation quantified. Horizontal line indicates activation cutoff from calibration data (**Supplementary Figure 1H**). Violin plots labelled with fraction activated, significance from Wilcoxon Test in R. Stars: * p ≤ 0.05 *** p ≤ 0.001 **** p ≤ 0.0001 ‘ns’ p > 0.05.

Our results thus far indicated two activation regimes – single cell response and whole culture response. We next wanted to probe the transition between these two regimes, and further investigate the role of culture density in this phenomenon. We used the FluidFM Cluster Stimulation protocol to stimulate a cluster of cells in the larger culture (**Figures 1D-F**). We focused on two C_max_ conditions in the FluidFM Cluster Stimulation experiments: one where the concentration of R848 was just below 100 nM in the center of the dish, and one where the concentration was just below 1 μM at the center of the dish (**Figure 3A**) (Mulder et. al, STAR Protocols, in press), using a stimulation time of 5 minutes. Before each experiment we confirmed the cells were in a resting state (**Supplementary Figure 3A**). A Cmax of 90 nM resulted in a smaller area of responding cells, in the region of 30 – 90 nM agonist concentration. A Cmax of 900 nM resulted in a larger area of responding cells, in the region of 100 – 900 nM agonist concentration (**Figure 3B**, **Supplementary Figure 3B**). Interestingly, increasing Cmax appeared to increase the activation threshold, restricting activation to a smaller area than expected if the threshold remained at a constant concentration (**Figure 3B**, **Supplementary Figure 3B**). This result suggested that there was more occurring with cellular activation than just the concentration alone; perhaps the surrounding cells influenced the responses of individual cells.

If cell to cell communication were impacting activation, we would expect to see a dependence on cell culture density. To determine if cell density was playing a role, we sub-categorized our data by cell density (**Supplementary Figure 1L**). Individual Cmax experiments were sorted into “sparse”, “moderate”, and “dense” cell density (**Supplementary Figure 1L**, **Figure 3B, C**). The “sparse” condition has cell density on par with that observed in tissue resident macrophages (36) and also the densities used in the single cell stimulation experiments.

For cluster stimulation with different culture densities, we calculated the fraction activated in the entire center region per experiment and categorized them by density (**Figure 3C**, **Supplementary Table 2**). Contrasting replicates with low cell density to those with high cell density, the difference between the two groups is significant for the Cmax = 90 nM experiments, and p = 0.07 for the Cmax = 900 nM experiments (**Figure 3C**). The decrease in percent activated was -21% +/- 6% and -20% +/- 5%, respectively.

While we categorized the culture images by average density, RAW264.7 cells tend to grow in clusters, rather than an even confluency. Therefore, even in experiments with a low overall cell density, there will be a mixture of isolated cells and clusters. We wanted to examine if the dependence on cell density correlated more with the average culture density or the local number of neighbors surrounding each cell. We determined the number of close neighbors for each cell by counting the number of other cells within ~2 cell lengths (18 μm) (Mulder et. al, STAR Protocols, in press). Looking just at cell density, we compared the response of cells in a sparse culture to a dense culture (**Supplementary Figure 3C**). The differences between the populations are highly significant for both Cmax values, as determined by 3-group permutation ANOVA (p < 0.0002 for both) and pairwise Wilcoxon test for sparse vs dense (p = 2e-18 and p = 9e-14 for Cmax = 90 nM and 900 nM, respectively). The effect sizes for the median FC in Nuc/Cyt NF-κB for these pairwise comparisons were -6% +/- 1% and -2.2% +/- 0.4% for Cmax = 90 nM and 900 nM, respectively. In both cases the cells were less activated at higher cell density. When we grouped the data by both number of neighbors and culture density (**Supplementary Figure 3C**) We found no significant difference between any of the groups with a 3-group permutation ANOVA, excepting the case of Cmax = 900 nM, spot density = sparse, comparing 0-1 neighbors with 2-3 neighbors (**Supplementary Figure 3D**). From this we concluded that differences in overall culture density were more important than the number of neighbors when cells were further apart. This implies that the signal regulating this process is likely distributed over longer distances.

To check if this phenomenon was specific to R848 stimulation or more general, we compared whole culture activation at different densities with three different TLR agonists: Lipopolysaccharide (LPS), a TLR4 agonist, Lipoteichoic Acid (LTA), a TLR2 agonist, and R848. We saw that activation did increase in more dense cultures over a range of agonists and concentrations, though the increases in fraction activated were smaller than for single cells versus a dense culture (**Figure 3D**).

In both cluster and whole culture stimulation, the collective NF-κB response is lower at higher cell density, suggesting a potential role for cell-to-cell communication in tuning macrophage sensitivity. The relative importance of whole culture density versus the hyper-local number of neighbors suggests a longer length and time scale for this communication.

### 3.4 Increased stimulation time restores activation in dense clusters

**Figure 4:**
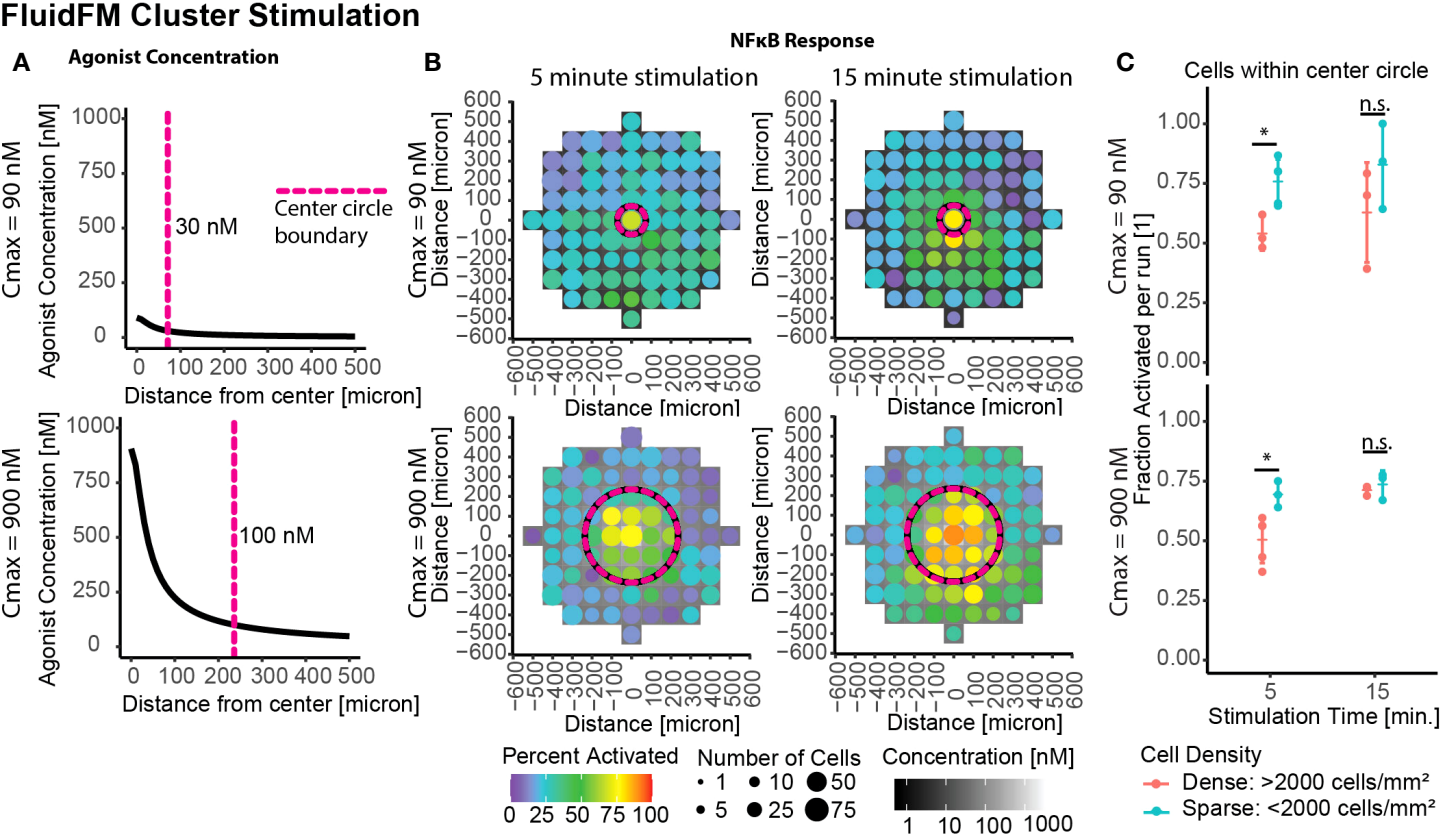
Increased stimulation time restores activation in dense clusters. Cluster stimulation with FluidFM at different stimulation times. **(A)** Concentration gradient during dispensing for C_max_ = 90 nM, 900 nM. Vertical dashed line marks where concentration drops below 30 nM (top) or 100 nM (bottom), cutoffs chosen based where activation drops below 50% for that stimulation condition (**Supplementary Figure 3A**). **(B)** Cluster targeting with FluidFM, densely plated cells, each plot one representative experiment (**Supplementary Figure 4A**). Top row C_max_ = 90 nM, bottom C_max_ = 900 nM, left and right columns 5- and 15-min stimulation time. Data binned in 100 μm x 100 μm squares, tile background is agonist concentration, point color is percent activated and point size is the number of cells per bin. Circular dashed line corresponds to dashed line in **(A)**. Negative control (top right) **(C)** Fraction activated in center per independent experiment, cutoff from calibration data (**Supplementary Figure 1H**). Grouped by stimulation condition (C_max_) and cell density. Significance from permutation ANOVA in R (Mulder et. al, STAR Protocols, in press). Stars indicate: * p ≤ 0.05, ‘ns’ p > 0.05.

A remaining question was if this decreased sensitivity was a permanent or transitory effect. Thus far we had focused on the shortest stimulation times possible with the FluidFM. To investigate the longevity of the phenomenon, we stimulated the clusters of cells for longer periods of time – analogous to previous culture experiments. Whole culture stimulation at 15 minutes or longer had an activation threshold around 100 nM or lower, similar to the single cell threshold for 5 minutes (**Figure 2D**). Further FluidFM Cluster Stimulation experiments were done with a 15-minute stimulation time to see if this effect extended to smaller clusters of cells as well. The same C_max_ of 90 and 900 nM are shown again as examples (**Figure 4A**, full data in **Supplementary Figure 4**).

In densely plated cells the longer stimulation times showed higher activation (**Figures 4B, C**). This difference was less pronounced in the sparse cells (**Figures 4B, C**). Increasing the stimulation time from 5 to 15 minutes caused the percent activated in densely plated cells to increase by 9%+/- 11% and 21% +/- 4% for Cmax = 90 nM and Cmax = 900 nM, respectively (**Figure 4C**). These results suggest that the density-dependent suppression of activation is most impactful for transitory stimuli (a few minutes), and if the stimulus persists, the majority of cells respond, resulting in a consistent sensitivity to signal. This combination of results explains (1) how this result would easily have been missed with previous experimental methods and (2) that the mechanism for this process must either occur in unstimulated culture or operate at timescales of <15 mins post-stimulation and over distances of multiple cell lengths.

### 3.5 Decreased activation is not due to decreased receptor expression or ligand uptake in dense culture

**Figure 5:**
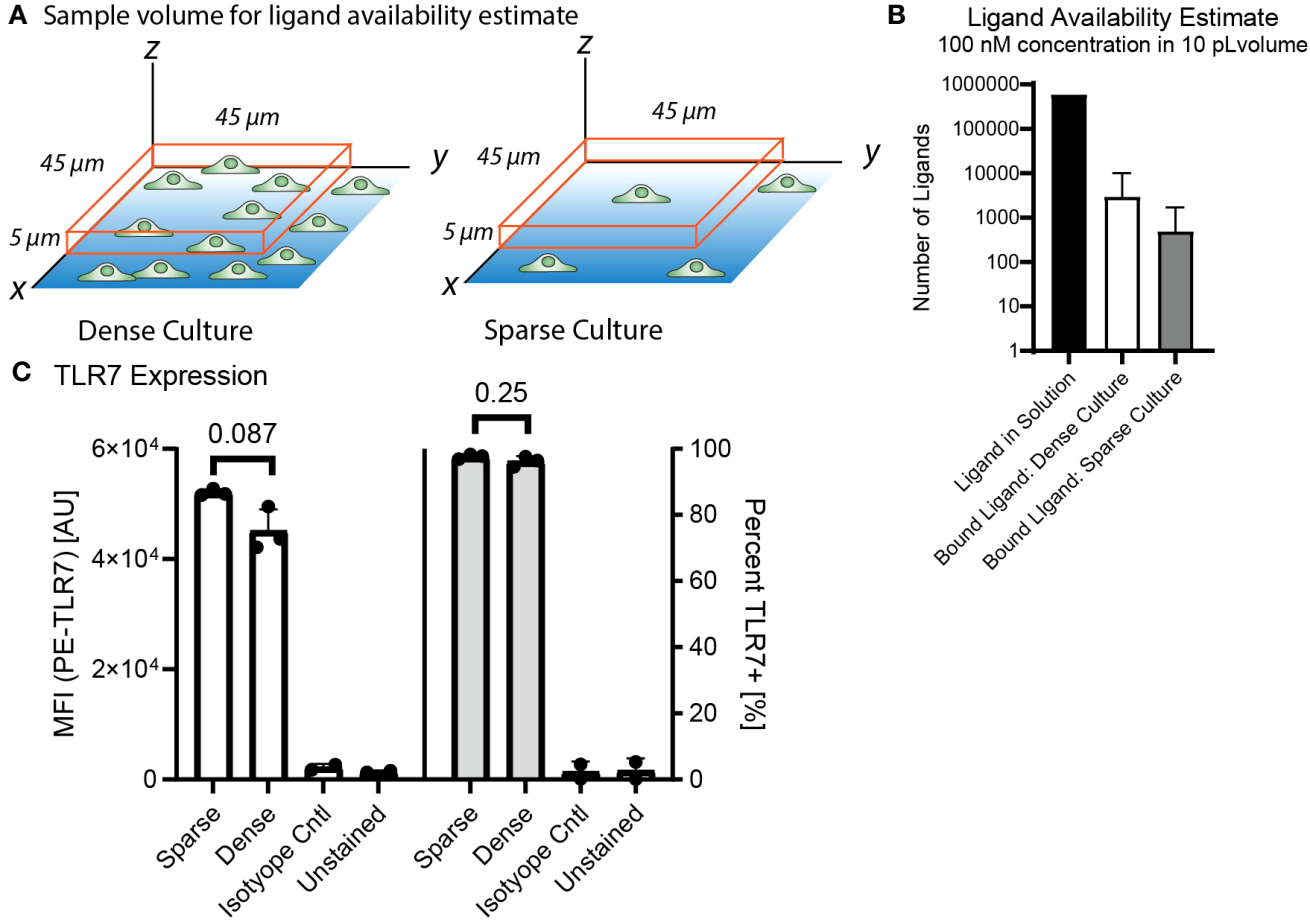
3.5 Decreased activation is not due to decreased receptor expression or ligand uptake in dense culture. **(A)** Diagram of sample volume in dense and sparse culture for ligand availability estimates. Cells on dish surface, orange box is sample volume, 5 microns high and 45 microns each side, encloses one cell in a sparse culture and one cluster of cells in a dense culture. **(B)** Ligand availability estimate in 10 pL volume at 100 nM concentration for dense and sparse cultures. Available ligand in solution vs estimated bound ligand in the volume (**Supplementary Table 3**). **(C)** TLR7 Expression from flow cytometry, compare dense vs sparse by median fluorescence intensity and percent TLR7+. Significance from unpaired t-test in R.

We hypothesized that this culture density dependent difference in NF-κB response was due to cell-to-cell communication based on culture density. However, we needed to rule out whether culture density could reduce activity through an alternative mechanism, such as local ligand uptake or lower TLR7 expression. To determine whether the cells in a dense culture could be impacting ligand availability for their neighbors through uptake, we estimated and compared ligand availability and receptor expression for a single cell or cluster of cells occupying the same volume under minimally activating stimulation of 100 nM R848 (**Supplementary Table 3**, **Figures 5A, B**). The result was that there should be tens to hundreds of times as many free ligands as bound ligands even in the dense culture, meaning ligand uptake by neighboring cells would not significantly affect the local agonist concentration. In addition, the ligand concentration is maintained by continuous dispensing from the FluidFM throughout stimulation so there is no decrease in ligand availability due to diffusion or uptake.

We also checked if culture density had any impact on TLR7 expression, and thus sensitivity to the TLR7 agonist R848. We performed flow cytometry to measure TLR7 expression by RAW 264.7 cells grown overnight at high and low density. We found no significant differences in TLR7 expression based on culture density (**Figure 5C**, **Supplementary Figure 5**).

Therefore, we think the observed inhibition of activation at greater cell densities is not due to ligand uptake or decreased receptor expression, and instead likely due to another communication mechanism. A cell density dependent modulation of agonist sensitivity and could have relevance to biological situations where macrophage density is increased.

## 4 Discussion

In summary, we observed the temporal and spatial characteristics of PRR signals influence the macrophage NF-κB response. The FluidFM allowed the unique ability to limit the temporal and spatial extent of the PRR signal to target single RAW 264.7 cells, small clusters, and larger collections of cells. We report that RAW 264.7 cells, in a controlled environment, deviate in their sensitivity to transitory (5 minute) PRR signals. Individual RAW 264.7 cells stimulated with PRR signals became more sensitive in windows under 5 mins when they do not experience the same signal concentration as the surrounding culture. This difference in individual cell responses led to a lower concentration threshold for NF-κB activation of single cells stimulated with the TLR agonist R848 compared to whole culture stimulation. The timescale of innate immune stimulation of a single macrophage in a biological situation is difficult to measure, but the observation of a minimum timescale for activation puts a boundary on a single cell’s ability to detect signs of infection. The fact that a larger population requires a longer stimulus to respond possibly indicates a mechanism for balancing sensitivity to small signals with preventing a damaging overreaction.

Our second question was how the surrounding cell culture influenced the sensitivity of individual cells. Since the FluidFM exposed neighboring cells to differing concentrations of PRR agonist, we wanted to understand how individual cells might integrate information from the larger unstimulated culture or local stimulated cluster. Cell-to-cell communication has been observed in related contexts - macrophages and other innate immune cells employ quorum licensing to regulate their state and quorum sensing to terminate inflammation (23, 38–41). In these cases, culture density during growth or after activation influence individual cell responses. For our experiments, we postulated that either the overall density of cells or the local number of cells would determine the sensitivity of the individually stimulated cells.

In our experiments we examined “sparse” cell densities (similar to the distribution of tissue resident macrophages) and “dense” cell densities (confluent or crowded). Simultaneously, we examined the “number of neighbors” (surrounding cells within a certain distance) in both conditions, to test whether local or global density was the dominant factor. We observed that the density of cells in culture determined the sensitivity of individuals to stimulation, with more dense cultures inhibiting individual cells from responding to lower concentrations of PRR agonist. The “number of neighbors” did not contribute to the phenomenon, leading us to conclude that the larger culture density was dominant in determining the response. Phrased colloquially, it’s not the neighbors on your street, but the overall population of your city that matters.

Upon providing evidence of a single cell to large-scale culture transition, and its relation to culture density, we sought to determine how this short-timescale phenomenon of 5 mins related to the more common experiment of stimulating macrophages for more than 30 mins in a culture dish. We performed a series of FluidFM experiments increasing the time of small cluster (tens to hundreds of cells) stimulations. We observed that the effect of alteration of sensitivity of clusters of cells is reduced after 15 mins of sustained PRR exposure. This result puts a temporal and spatial limit on potential mechanisms for the decreased activation of densely cultured cells exposed to transitory PRR signals, and suggests that the mechanism of cellular communication must occur at a similar time scale or precede the appearance of the PRR agonist. We also tested alternative mechanisms besides cell-to-cell communication and concluded that neither ligand uptake nor differences in receptor expression would explain our observations. It is also possible that our observations are part of a program of tolerance due to chronic stimulation from contamination, though we have attempted to prevent contamination and monitor for signs of activation before each experiment. The next step of this research is an investigation into potential mechanisms that could explain these changes in activation threshold. If such mechanism(s) were identified, they could be used to tune macrophage sensitivity in contexts such as dose-sparing for vaccines or tamping down excess inflammation.

While we focused on NF-κB activation in these studies, this approach could be expanded to other measures of macrophage activation. An initial NF-κB response does not necessarily lead to a downstream action such as cytokine release or phagocytic activity. Further studies of these responses would need to be done to assess the functional importance of our observed differences in NF-κB response to short-timescale stimuli. These results also open up more questions about the dynamics of TLR activation and response, potential collective coordination of the response, and the role of the local cell environment. The FluidFM provides a valuable tool for this research because it can provide both persistent and transitory stimuli, as well as spatially varying gradients, with a preservation of any secreted factors present before stimulation. In this way it approximates the situation of quiescent cells sparsely distributed encountering localized and/or transitory signals of infection. The cell culture environment can be varied to explore its effect on macrophage sensitivity to these signals. Our experimental approach can be used to study a variety of cell types, environments, stimuli, and responses to explore further questions of single immune cell responses.

## Data availability statement

The original contributions presented in the study are included in the article/**Supplementary Materials**. Further inquiries can be directed to the corresponding author. All data and code for this manuscript can be found at Mendeley Data, DOI: 10.17632/mfyddz6n8k.3.

## Ethics statement

Ethical approval was not required for the studies on animals in accordance with the local legislation and institutional requirements because only commercially available established cell lines were used.

## Author contributions

EM: Conceptualization, Data curation, Formal analysis, Investigation, Methodology, Validation, Visualization, Writing – original draft, Writing – review & editing. BM: Conceptualization, Methodology, Writing – review & editing. JD: Investigation, Methodology, Writing – review & editing. RS: Conceptualization, Methodology, Writing – review & editing. AE: Conceptualization, Funding acquisition, Project administration, Supervision, Writing – review & editing.
