# Supplementary material for: Evidence of collective influence in innate sensing using fluidic force microscopy
Source: Front Immunol. 2024 Jan 23;15:1340384. doi: 10.3389/fimmu.2024.1340384 (PMC10844469; doi:10.3389/fimmu.2024.1340384)
Supplement: Supplementary file 1 [file DataSheet_1.docx]

Supplementary Material


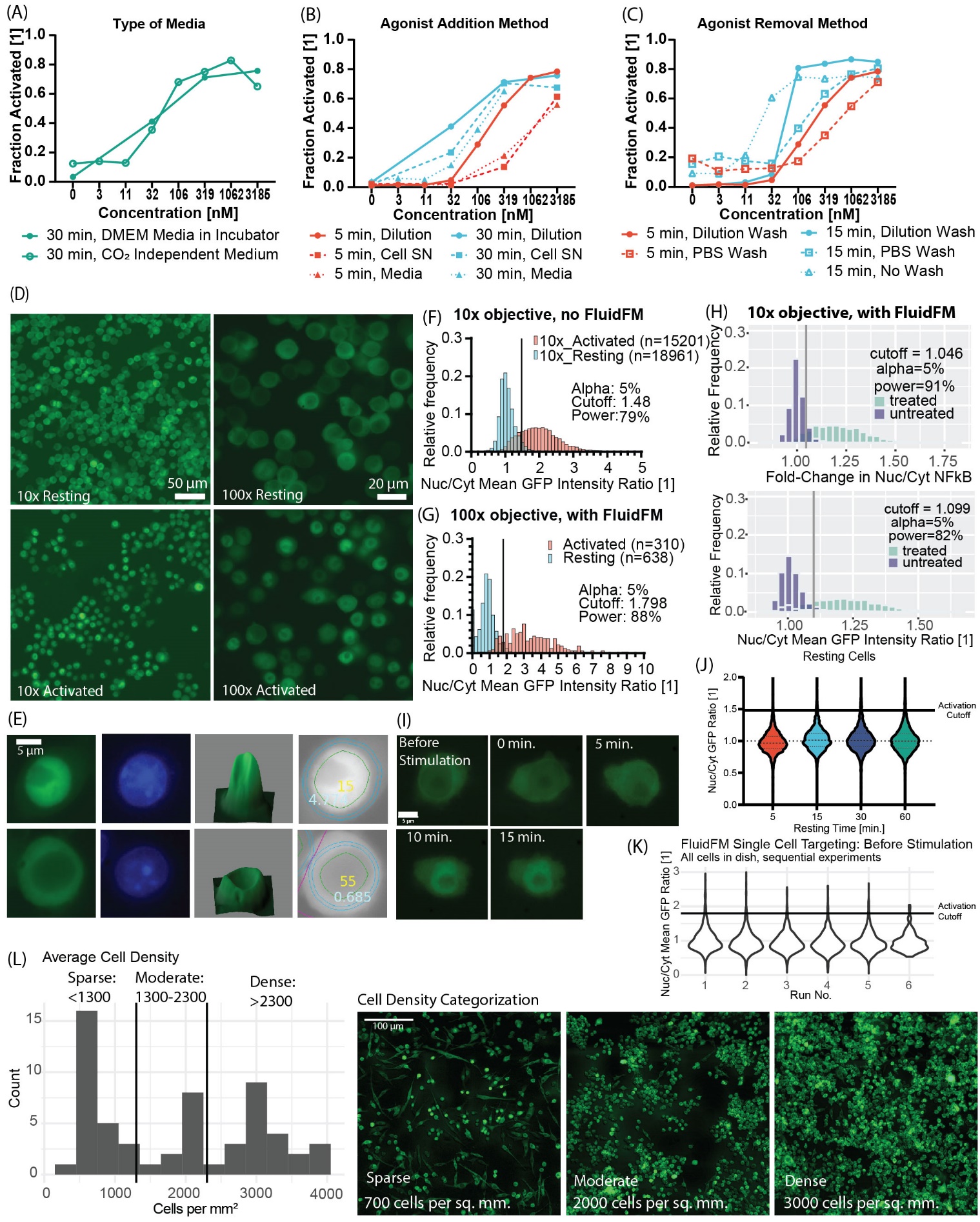


**Supplementary Figure 1. (A-C)** Whole culture stimulation for short timescales method development. Dose curves of concentration R848 vs fraction activated in well. Varied assay parameters of **(A)** type of media, **(B)** agonist addition method, and **(C)** agonist removal method. Color indicates stimulation time (red 5 min, blue 15 min, green 30 min), point fill indicates media type (solid: DMEM media at 37°C and 5% CO_2_, open: CO_2_ Independent Medium at 37°C), point shape and line style indicate agonist addition or removal method.  **(D)** Representative images of calibration data using NF-κB reporter cells. Left: 10x images of resting (top) and activated (bottom) cells, scale is 50 microns. Right: 100x images of activated cells before (top) and after (bottom) stimulation. Scale is 20 microns. **(E)** Cell image segmentation for quantification. Top row: activated macrophage. Bottom row: unactivated macrophage. Left columns: raw GFP fluorescence and hoechst nuclear stain images. Center: 3D surface plot from ImageJ showing pixel intensity. The cytoplasm that appears uniformly flat in brightness in the raw images is in fact highly sloped in actual pixel intensities as shown by the surface plot. Right column: ROIs for quantification overlaid on the GFP intensity image, labeled in yellow with a cell number and blue with the nuclear/cytoplasmic GFP ratio. The nuclear ROI is outlined in green, the cytoplasmic ring ROI is outlined in blue, and the pink line shows the segmentation boundary between two cells. The cytoplasmic ring captures the region of highest intensity for the cytoplasm, giving the greatest accuracy in distinguishing between activated and unactivated macrophages. **(F-H)** Quantified data from **(C)**, used to determine activation cutoffs for **(F)** 10x images without the FluidFM in the light-path (whole culture activation data), **(G)** 100x images of cells (single cell activation data), and **(H)** 10x images of cells with FluidFM in the light-path (cluster activation data). Histograms of resting and activated populations with activation cutoff shown, annotated with cutoff value “cutoff”, percent negative population above cutoff “alpha”, and percent positive population above cutoff “power.” Top panel: fold-change measurement, bottom panel: single timepoint measurement. **(I)** Example time-course of target macrophage under single cell stimulation with R848: before stimulation, and at 0, 5, 10, and 15 minutes relative to the start of stimulation. The 15 minute timepoint was chosen as the readout time for these experiments. **(J)** Resting cells Nuc/Cyt mean GFP ratio over time. Untreated RAW 264.7 cells incubated for 5, 15, 30, and 60 minutes at 37°C and 5% CO_2_, fixed, then imaged. Horizontal line is activation cutoff from calibration data (F). **(L)** Resting cells Nuc/Cyt mean GFP ratio over sequential experiments in same dish, data taken before stimulation. Same data as in Figure S2A but grouped by run number instead of experimental condition. Since each run takes about 1 hr., the run number is a proxy for time the cells have been on the microscope before being used. Horizontal line is activation cutoff from (G). **(K)** Categorization of cell cultures into 3 density categories. Left: histogram of culture densities from cluster stimulation experiments, cutoffs at 1300 and 2300 cell/mm^2^ determined from distribution. Right: example images from each category, green fluorescence is NF-κB-GFP, scalebar 100 microns.


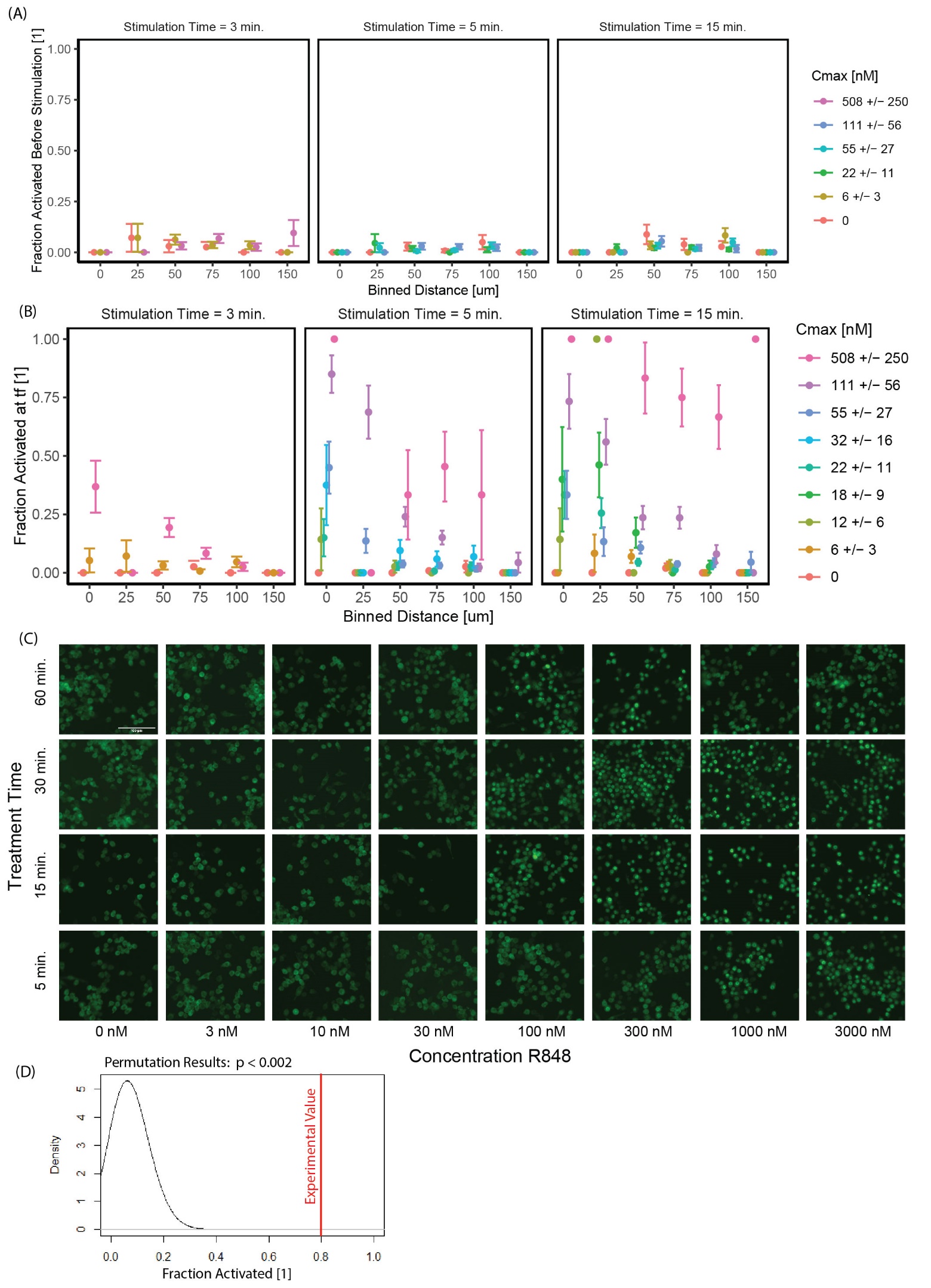


**Figure S2. (A-B)** FluidFM single cell targeting experiments. Fraction activated vs distance from dispensing point, colors are concentration at target cell position (Cmax), stimulation times (left to right) are 3, 5, and 15 minutes. Uncertainty in fraction activated from bootstrap method (Mulder et.al, *STAR Protocols,* in press). **(A)** Cells are in resting state before stimulation with FluidFM. **(B)** All FluidFM single cell targeting experiments, including those not shown in Figure 2C. Target cell results summarized in Table S1. **(C)** Sample images for the data shown in Figure 2D. GFP-tagged NF-κB fluorescence. Images are organized horizontally by increasing concentration R848, and vertically by treatment time. Scalebar 100 microns. **(D)** Results of permutation method for statistical significance of the difference in fraction activated, used to determine statistical significance for the comparison of single cell and group activation in Figure 2E (Mulder et.al, *STAR Protocols,* in press).

**Table S1: FluidFM Single Cell Targeting: Target Cell Outcomes.**

| Treatment Time | C_0_ [μM] | Estimated C_max_ [nM] | Uncertainty in Cmax [nM] | # activated by t_f_ cutoff | Total runs |
| --- | --- | --- | --- | --- | --- |
| 3 min. | 0 | 0 | -- | 0 | 5 |
| 3 min. | 0.3 | 6 | 3 | 1 | 19 |
| 3 min. | 25 | 508 | 250 | 7 | 19 |
| 5 min. | 0 | 0 | -- | 0 | 5 |
| 5 min. | 0.6 | 12 | 6 | 1 | 7 |
| 5 min. | 1.1 | 22 | 11 | 3 | 20 |
| 5 min. | 1.6 | 33 | 16 | 3 | 8 |
| 5 min. | 2.8 | 55 | 27 | 9 | 20 |
| 5 min. | 5.6 | 111 | 56 | 17 | 20 |
| 15 min. | 0 | 0 | -- | 0 | 5 |
| 15 min. | 0.3 | 6 | 3 | 0 | 15 |
| 15 min. | 0.6 | 12 | 6 | 1 | 7 |
| 15 min. | 0.9 | 18 | 9 | 2 | 5 |
| 15 min. | 1.1 | 22 | 11 | 7 | 21 |
| 15 min. | 2.8 | 55 | 27 | 7 | 21 |

**Table S1.** Results of all FluidFM Single Cell Stimulation experiments. Treatment time: duration of FluidFM stimulation. C_0_: the concentration loaded into the probe. C_max_ : the estimated concentration below the dispensing point. # activated by t_f_ cutoff: the number of target cells that were activated according to the cutoff for final timepoint activation (Figure S1G). Total runs: the total number of replicates for that condition.


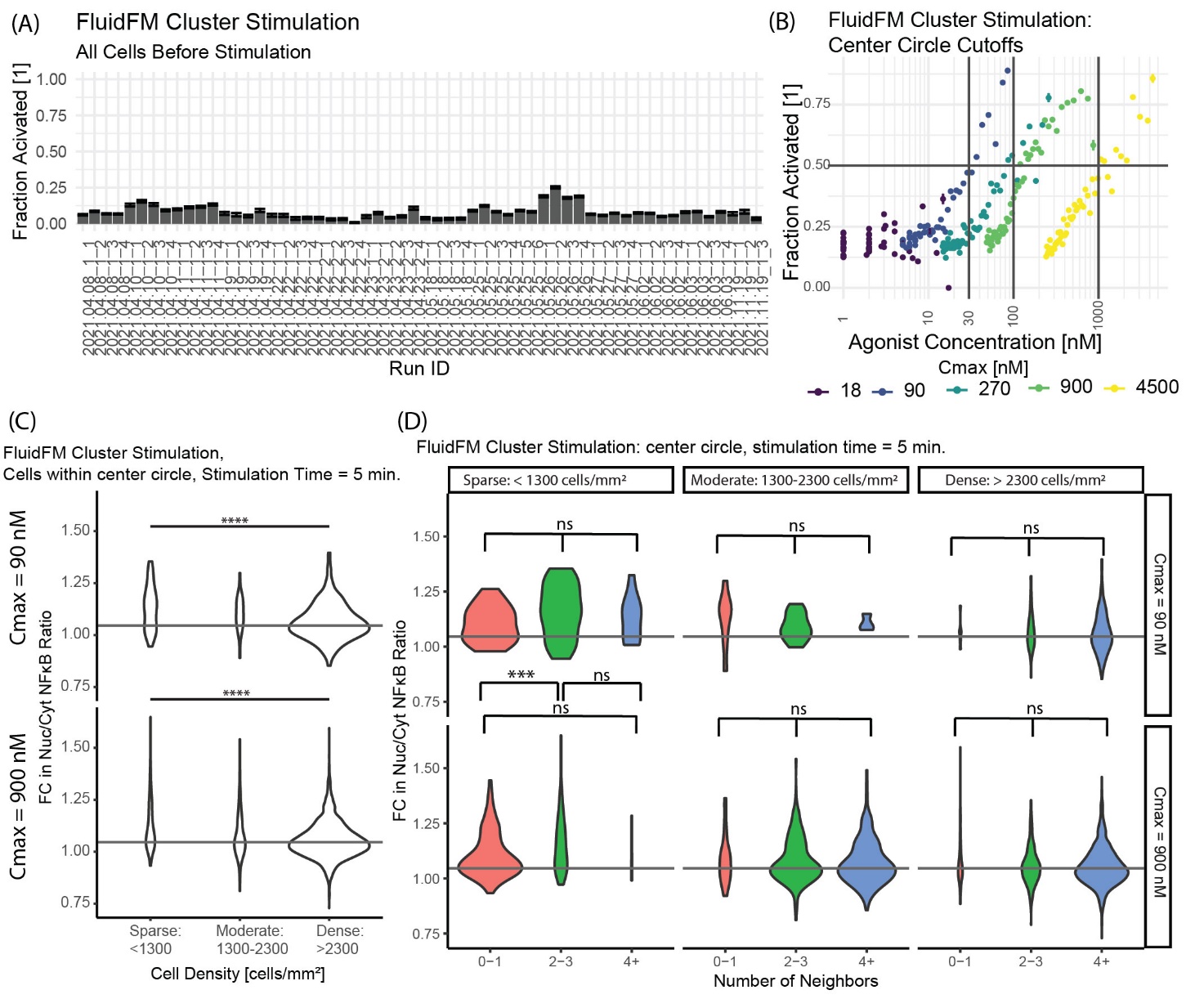


**Figure S3. (A)** Cell state before stimulation. Individual runs on x axis, fraction activated in field of view on y axis, error bars uncertainty in fraction activated from bootstrap method (Mulder et.al, *STAR Protocols,* in press). Activation cutoff from calibration data. **(B)** Method for choosing center circle cutoff locations (Figure 3B, 4B). FluidFM cluster stimulation data, binned by distance (10 μm bin-width), plotted by concentration at that distance versus fraction activated, grouped by Cmax. Stimulation time 5 min. Horizontal line at 50% activated, vertical lines at 30, 100, and 1000 nM, where the curves each intersect the 50% line. **(C)** Fold-change in Nuc/Cyt NF-κB ratio vs average cell density and number of neighbors (defined as number of other nuclei within 18 microns radius). Horizontal line is activation threshold (Figure S1H). Significance determined by 3-group permutation ANOVA in R and pairwise comparisons with Wilcoxon test in R (Mulder et.al, *STAR Protocols,* in press). **(D)** Comparison of number of neighbors vs average cell density to determine which is the more important factor in activation. Horizontal axis is the number of individual cell neighbors, grouped into 3 categories: 0-1, 2-3, and 4+. Vertical axis is the FC in Nuc/Cyt NF-κB ratio between the initial and final timepoints. Horizontal line shows the activation cutoff as determined from calibration data (Fig S1H). Color is number of neighbors. Plots are grouped in columns by average cell density of the entire dataset: sparse, moderate, and dense. Rows are the Cmax values of 90 and 900 nM, corresponding to the data shown in Figure 3 (all of these data are 5-minute stimulation time). Statistical significance determined by 3-group permutation ANOVA in R, followed by pairwise Wilcoxon test in R if a significant difference was found (Mulder et.al, *STAR Protocols,* in press). Stars indicate: *** p ≤ 0.001 **** p ≤ 0.0001 ‘ns’ p > 0.05


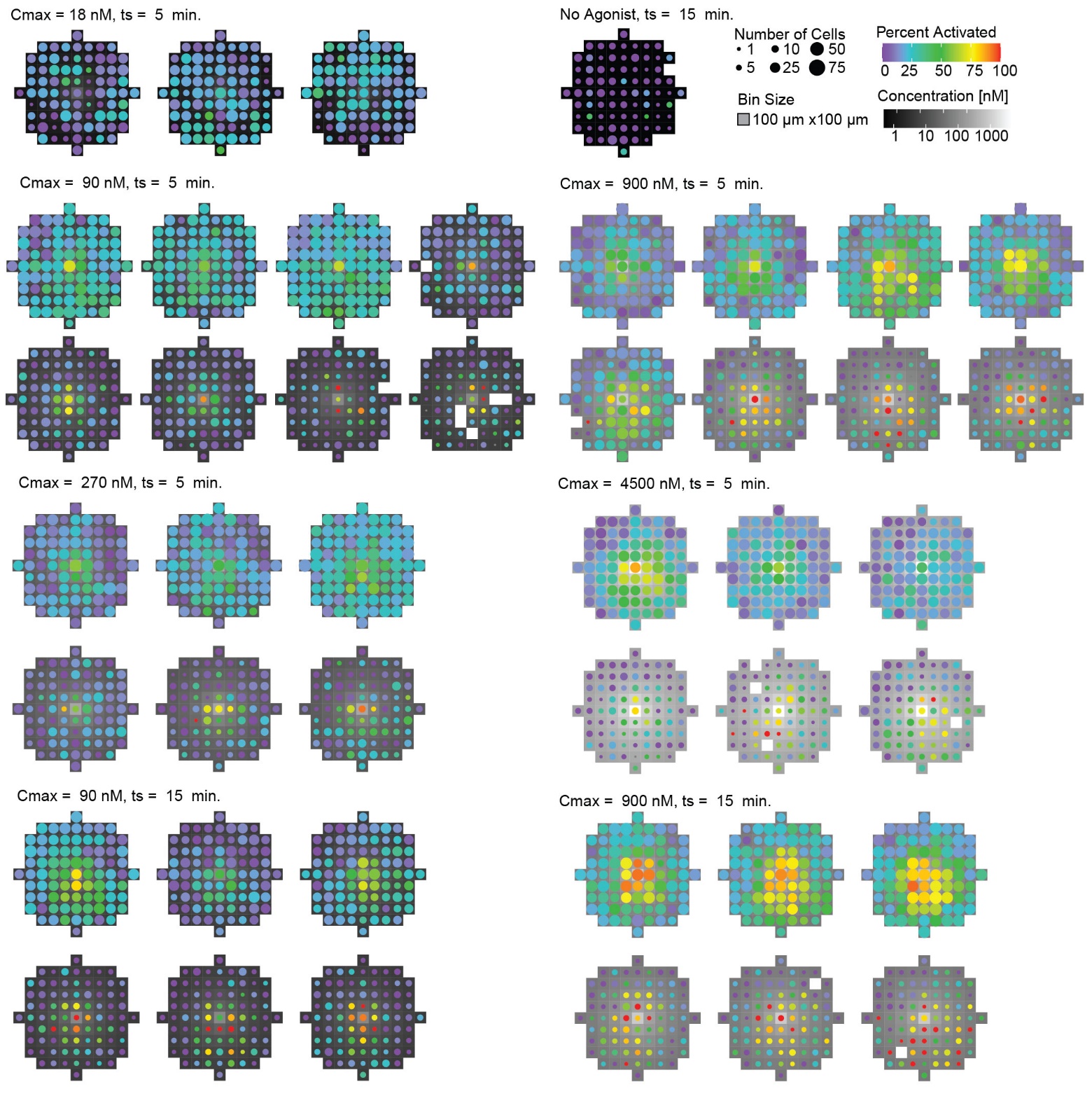


**Figure S4.** FluidFM cluster stimulation, all datasets. Each plot is an individual run, runs are grouped by stimulation condition (Cmax = R848 concentration in the center of the spot, ts = stimulation time) and ordered by culture density (decreasing). Data shown is percent activated (cutoff for fold-change in Nuc/Cyt NF-κB from initial to final timepoint), binned by cell location in the dish (bins are 100 microns wide). Tile greyscale is concentration at that location in the dish (Mulder et.al. 2023 *submitted*). Point size is scaled to number of cells in that bin (a measure of local cell density). Point color shows percent activation in that bin (Figure S1H). Results summarized in Table S2.

**Table S2:** **FluidFM Cluster Targeting Results Summary**

| **C_0_ [uM]** | **C_max_ [nM]** | **Time [min]** | **Density [cells/mm^2^]** | **Center Threshold [nM]** | **% Activated** |
| --- | --- | --- | --- | --- | --- |
| 0 | 0 | 15 | 500 | 0 | 8% |
| 0.064 | 18 | 5 | 2000 | 0 | 15% |
| 0.064 | 18 | 5 | 2100 | 0 | 19% |
| 0.064 | 18 | 5 | 2200 | 0 | 19% |
| 0.318 | 90 | 5 | 500 | 30 | 67% |
| 0.318 | 90 | 5 | 600 | 30 | 80% |
| 0.318 | 90 | 5 | 1200 | 30 | 66% |
| 0.318 | 90 | 5 | 1200 | 30 | 87% |
| 0.318 | 90 | 5 | 1700 | 30 | 80% |
| 0.318 | 90 | 5 | 2800 | 30 | 48% |
| 0.318 | 90 | 5 | 3500 | 30 | 62% |
| 0.318 | 90 | 5 | 3800 | 30 | 52% |
| 0.318 | 90 | 15 | 700 | 30 | 100% |
| 0.318 | 90 | 15 | 700 | 30 | 64% |
| 0.318 | 90 | 15 | 800 | 30 | 84% |
| 0.318 | 90 | 15 | 2200 | 30 | 39% |
| 0.318 | 90 | 15 | 2200 | 30 | 70% |
| 0.318 | 90 | 15 | 3000 | 30 | 79% |
| 0.955 | 270 | 5 | 1000 | 100 | 94% |
| 0.955 | 270 | 5 | 1100 | 100 | 74% |
| 0.955 | 270 | 5 | 1600 | 100 | 50% |
| 0.955 | 270 | 5 | 2800 | 100 | 56% |
| 0.955 | 270 | 5 | 3300 | 100 | 43% |
| 0.955 | 270 | 5 | 3900 | 100 | 51% |
| 3.185 | 900 | 5 | 600 | 100 | 64% |
| 3.185 | 900 | 5 | 600 | 100 | 75% |
| 3.185 | 900 | 5 | 700 | 100 | 69% |
| 3.185 | 900 | 5 | 2000 | 100 | 56% |
| 3.185 | 900 | 5 | 3000 | 100 | 60% |
| 3.185 | 900 | 5 | 3000 | 100 | 56% |
| 3.185 | 900 | 5 | 3100 | 100 | 37% |
| 3.185 | 900 | 5 | 3100 | 100 | 43% |
| 3.185 | 900 | 15 | 400 | 100 | 67% |
| 3.185 | 900 | 15 | 500 | 100 | 78% |
| 3.185 | 900 | 15 | 500 | 100 | 76% |
| 3.185 | 900 | 15 | 3000 | 100 | 73% |
| 3.185 | 900 | 15 | 3300 | 100 | 69% |
| 3.185 | 900 | 15 | 3800 | 100 | 72% |
| 15.924 | 4500 | 5 | 500 | 1000 | 79% |
| 15.924 | 4500 | 5 | 700 | 1000 | 75% |
| 15.924 | 4500 | 5 | 800 | 1000 | 65% |
| 15.924 | 4500 | 5 | 2500 | 1000 | 32% |
| 15.924 | 4500 | 5 | 3000 | 1000 | 74% |
| 15.924 | 4500 | 5 | 3000 | 1000 | 49% |

**Table S2.** C_0_: the concentration loaded into the probe. C_max_ : the estimated concentration below the dispensing point. Time: stimulation time. Density: average cell density in the field of view. Center Threshold: cutoff for filtering “cells in center circle”, determined by plotting fraction activated vs concentration (cells grouped together in bins of 10 microns distance from dispensing point) and finding the approximate point at which the fraction activated dropped below 50% for each C_max_. Percent Activated: percent activated within center circle for each run.


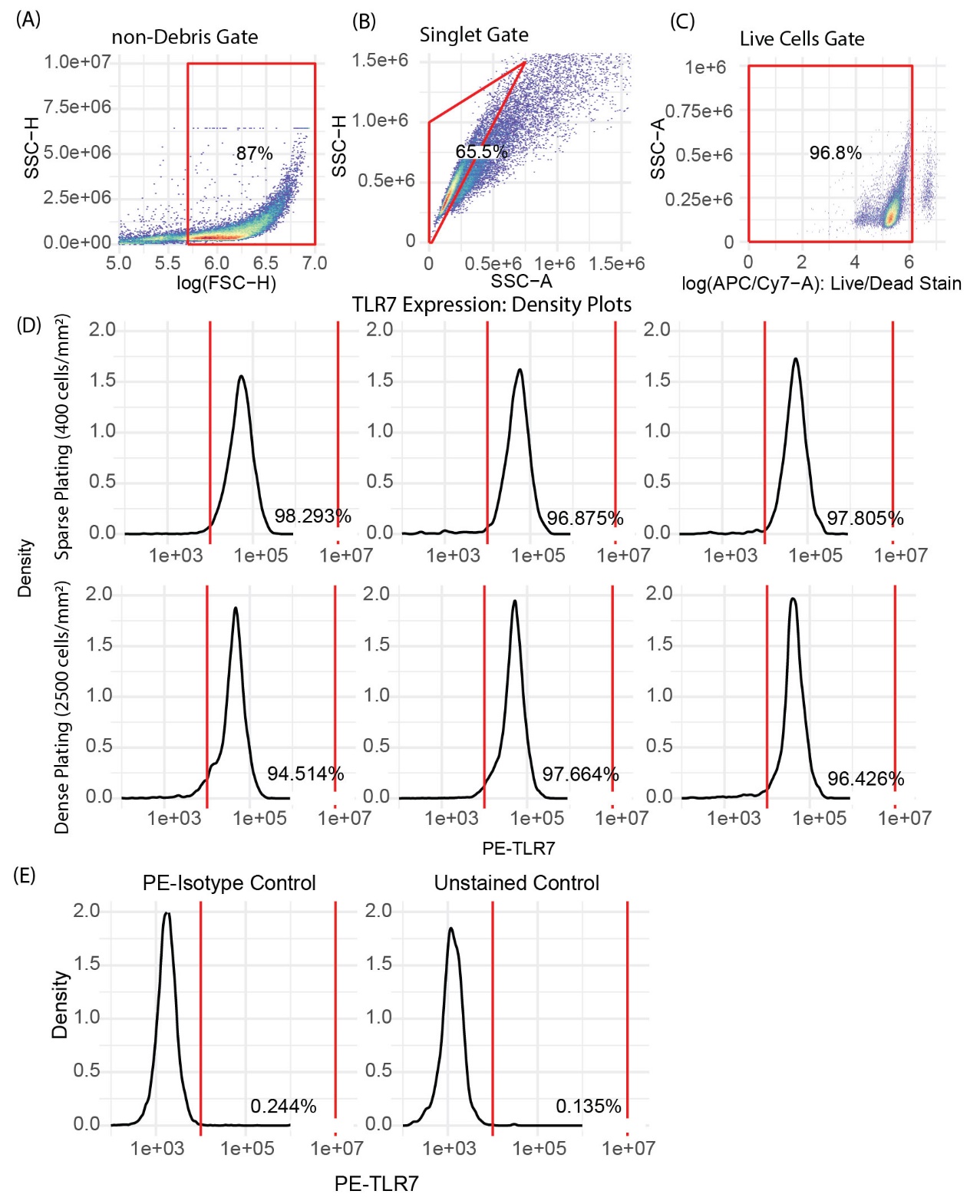


**Figure S5.** Flow cytometry of TLR7 expression for macrophages cultured at high and low density (plated at 400 cells/mm^2^ and 2500 cells/mm^2^ respectively, then grown overnight). **(A-C)** Gating strategy (example plots), left to right, non-debris (high FSC), singlets (lower SSC-A for same SSC-H), live cells (lower fluorescence live/dead reactive dye). **(D)** PE-TLR7 Ab fluorescence density plots. Top row, sparse plating, bottom row dense plating. Red lines: gate for TLR7+. **(E)** Isotype control and unstained control. No non-specific staining.

**Table S3: Estimate of ligand availability in FluidFM Cluster Stimulation.**

| **How many R848 ligands are available?** | | | | |
| --- | --- | --- | --- | --- |
|  | **Dense Culture** | **Sparse Culture** |  |  |
| Minimum activating concentration 100 nM | 10^-7^ M | 10^-7^ M | This paper |  |
| Sample volume | 10 pL | 10 pL | Figure S5A |  |
| Cell Density | 3000 cells / mm^2^ | 500 cells / mm^2^ | This paper |  |
| Cells / sample | 6 | 1 | Cell density*sample area (Figure S5A) |  |
| *Ligands / sample* | 6 x 10^5^ | 6 x 10^5^ | Ligand conc. * sample volume * Avogadro’s Number |  |
| **How many TLR7 receptors are present?** | | | | |
| Number of single receptor type on single mouse endothelial cell | 10^3^-10^4^ | 10^3^-10^4^ | Imoukhuede, P.I. and Popel A.S. (2012) *PLoS ONE* |  |
| Estimated number of TLR7 per single macrophage | 10^3^-10^4^ | 10^3^-10^4^ | Mouse macrophage similar size to mouse endothelial cell |  |
| *TLR7 in sample volume* | 6 x 10^3^- 6 x 10^4^ | 10^3^ - 10^4^ | # Cells * Receptors/Cell |  |
| **How much ligand is bound at 100 nM concentration?** | | | | |
| R848-TLR7 k_d_ | 4.9 x 10^-7^ M | 4.9 x 10^-7^ M | Zhang et.al. (2016) *Immunity* |  |
| Fractional occupancy at 100 nM | 0.17 | 0.17 | [L] / ( [L] + k_d_ )  Salahudeen and Nishtala (2017) *Saudi Pharm J* |  |
| *Amount of bound ligand at 100 nM* | 10^3^- 10^4^ | 1.7 x 10^2^ - 1.7 x 10^3^ | Fractional occupancy * total receptors |  |
| **What is the ratio of available ligand to bound ligand?** | | | | |
| *Available / bound ligand* | 60 - 600 | 350 – 3500 | Ligands in volume / Bound ligand in volume |  |

**Table S3.** Ratio of available ligand to bound ligand in FluidFM Cluster Stimulation. First column: relevant quantities, second column: estimates of their values for dense culture, third column: estimates of values for sparse culture, fourth column: source for these values or method of calculating them. Number of TLR7 receptors estimated from measurements of a single receptor type on mouse cells of a similar size measured by Imoukhuede and Popel, in the absence of direct measurements of TLR7 abundance. R848 affinity for TLR7 measured by Zhang et.al. Fractional occupancy calculated as described by Salahudeen and Nishtala. Sample volume used to calculate ligand quantities taken to be a box occupied by 1 cell in sparse culture and 1 cluster in dense culture (Figure 5A). Even at the highest cell density, there is several times more available ligand than bound ligand.
